# Immunomodulatory Effect of *Cordyceps militaris* Polysaccharide on RAW 264.7 Macrophages by Regulating MAPK Signaling Pathways

**DOI:** 10.3390/molecules29143408

**Published:** 2024-07-20

**Authors:** Yan Liu, Jiayi Yang, Zhijian Guo, Qizhang Li, Lida Zhang, Lingxia Zhao, Xuanwei Zhou

**Affiliations:** 1 School of Agriculture and Biology, Shanghai Jiao Tong University, Shanghai 200240, China; liuyan8468@sjtu.edu.cn (Y.L.); silvan1010@sjtu.edu.cn (J.Y.); gzj19991006@sjtu.edu.cn (Z.G.);; 2 Engineering Research Center of Therapeutic Antibody (Ministry of Education), Shanghai Jiao Tong University, Shanghai 200240, China; 3 Innovative Drug R&D Center, College of Life Sciences, Huaibei Normal University, Huaibei 235000, China

**Keywords:** *Cordyceps militaris*, polysaccharide, immunomodulatory activity

## Abstract

Polysaccharide is one of the principal bioactive components found in medicinal mushrooms and has been proven to enhance host immunity. However, the possible mechanism of immunomodulatory activity of *Cordyceps militaris* polysaccharide is not fully understood. Hot water extraction and alcohol precipitation, DEAE-Sephadex A-25 chromatography, and Sephadex G-100 chromatography were used to isolate polysaccharide from *C. militaris*. A high-molecular-weight polysaccharide isolated from *C. militaris* was designated as HCMP, which had an Mw of 6.18 × 10^5^ Da and was composed of arabinose, galactose, glucose, mannose, and xylose in a mole ratio of 2.00:8.01:72.54:15.98:1.02. The polysaccharide content of HCMP was 91.2% ± 0.16. The test in vitro showed that HCMP activated mouse macrophage RAW 264.7 cells by enhancing phagocytosis and NO production, and by regulating mRNA expressions of inflammation-related molecules in RAW 264.7 cells. Western blotting revealed that HCMP induced the phosphorylation of mitogen-activated protein kinases (MAPKs). Moreover, using inhibitors of MAPKs decreased the mRNA levels of inflammation-related molecules induced by HCMP. These data evidenced that the immunomodulatory effect of HCMP on RAW 264.7 macrophages was mediated via the MAPK signaling pathway. These findings suggested that HCMP could be developed as a potent immunomodulatory agent for use in functional foods and dietary supplements.

## 1. Introduction

Immunomodulation is a vital strategy to raise the body’s defense and recently has gained a broad interest. Using immunomodulators to enhance host defenses has proved to be an effective strategy for combating infectious diseases. Macrophages are the first to recognize external stimuli. Upon interacting with external molecular triggers, pattern recognition receptors (PRRs) on the surface of macrophages can initiate intracellular signaling cascades—for instance, the activation of mitogen-activated protein kinases (MAPKs), including extracellular-signal-regulated kinase (ERK), Jun-N-terminal kinase (JNK), and p38 mitogen-activated protein kinase (MAPK). The activation process mediates the transcription of cytokines, such as tumor necrosis factor-α (TNF-α) and interleukins (ILs) [1], thereby actively participating in the orchestration of inflammatory responses [2].

*Cordyceps militaris* is one kind of *Cordyceps* species, distributed at altitudes surpassing 2000 m globally. *C. militaris* is feasible for artificial cultivation and possesses comparable chemical properties and pharmacological effects similar to *Ophiocordyceps sinensis*, which is a very rare species grown on the Tibet Plateau and needs to be protected for sustainable use [3]. *C. militaris* exhibits a spectrum of pharmacological effects, including anti-inflammatory, anti-tumor, antioxidant, lipid-lowering, hypoglycemic, etc., which are associated with its impact on immune responses [4,5].

Polysaccharide is one of the principal bioactive components found in medicinal mushrooms and has proved able to enhance host immune system functions [6,7], such as hydroxyethylated *Hericium erinaceus* [8] and *Ganoderma applanatum* [9]. Polysaccharide mainly exists in the cell wall, which can be broken by hot water without destroying the polysaccharide structure, and it is easy to extract. Based on the different ionic properties and molecular weight distributions of polysaccharides, polysaccharides can be isolated from the extracts by DEAE-Sephadex A-25 chromatography and Sephadex G-100 chromatography. Some studies have shown that *C. militaris* polysaccharide has immunomodulatory activity; however, there are few studies on its underlying immunomodulatory mechanism.

In this study, a high-Mw polysaccharide beyond the Mw range of active polysaccharides in previous studies was isolated from the fruiting body of *C. militaris* (designated as HCMP). The immunomodulatory activity of HCMP and its underlying mechanism was investigated. This study demonstrates valuable opinions for understanding the potential role of *C. militaris* polysaccharide in immune responses and establishes an experimental foundation for considering HCMP as an immunomodulatory agent. Still, following this study, related animal experiments on the immunomodulatory activity of HCMP are warranted, which is also a limitation of this study and a point for future research.

## 2. Results and Discussion

### 2.1. Isolation of HCMP

**Figure 1:**
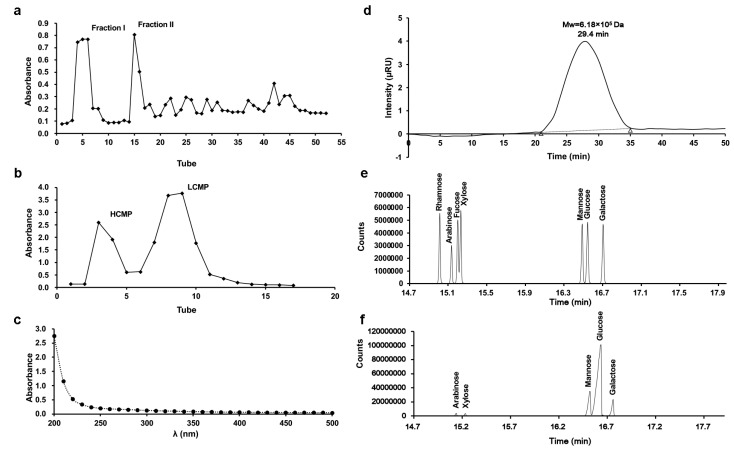
Isolation and characteristics of HCMP. (**a**) Elution profile of crude polysaccharide of *C. militaris* on DEAE-Sephadex A-25 column. (**b**) Elution profile of Fraction I on Sephadex G-100 column. (**c**) UV spectrum of HCMP. (**d**) Molecular weight profile of HCMP determined by gel permeation chromatography. (**e**) Gas chromatograms of the standard monosaccharide of the mixture. (**f**) Gas chromatograms of HCMP.

Two fractions, Fraction I and Fraction II, were derived from *C. militaris* crude polysaccharide through DEAE-Sephadex A-25 chromatography (Figure 1a). Subsequently, Fraction I underwent subsequent separation via Sephadex G-100 chromatography, generating two distinct components (Figure 1b). According to the separation mechanism of glucan gel column chromatography, the high-molecular-weight component is eluted first, and the low molecular weight component is eluted later. The first elution with higher Mw was collected, freeze-dried, and stored, and it was designated as HCMP. There were no absorptions at 260 nm and 280 nm in the UV spectrum, indicating that HCMP was free of protein and nucleic acid (Figure 1c). The polysaccharide content of HCMP was 91.2% ± 0.16.

### 2.2. Mw Distribution and Monosaccharide Composition of HCMP

The profile of gel permeation chromatography indicated that the average Mw of HCMP was 6.18 × 10^5^ Da (Figure 1d). GC analysis revealed that HCMP consisted of arabinose, galactose, glucose, mannose, and xylose, with a mole ratio of 2.00:8.01:72.54:15.98:1.02 (Figure 1f), after comparing with the position of monosaccharides in the mixed standard (Figure 1e). HCMP was rich in glucose, mannose, and galactose.

The Mw of polysaccharide is a crucial factor affecting its bioactivity performance [10]. Previous studies have reported a range of Mw from 1.273 × 10^3^ to 4.36 × 10^4^ Da of bioactive polysaccharide derived from *C. militaris* [11,12]. Recent research by Zhang et al. [13] presented that the Mw of the polysaccharide SDQCP-1 with antioxidant and immunomodulatory activities from *C. militaris* was 1.93 × 10^5^ Da. Our data evidenced that the average Mw of HCMP (6.18 × 10^5^ Da) was beyond the Mw range of previous studies, which indicated that HCMP was a novel polysaccharide isolated from *C. militaris.* It has been reported that polysaccharide with higher Mw often has higher biological bioactivity [11,12]. Thus, it was deduced that HCMP might have high biological activity.

The bioactivity of polysaccharides is related to their monosaccharide composition [14]. A large number of natural polysaccharides are reported to be ligands of Toll-like receptors (TLRs), such as TLR4 [15]. Glucose, galactose, and mannose are the most common in all TLR4-related polysaccharides [16]. For instance, glucan with branches of glucosyl residues showed immunomodulatory activity to induce IL-12 p40 and TNF-α production in macrophages via TLR4 signaling [17]. Mannose was found to enhance naive CD4+ T cell differentiation to Treg cells and enhance immunomodulation [18]. The substantial presence of galactose, glucose, and mannose in HCMP may facilitate TLR4 recognition in RAW 264.7 macrophages, thereby potentially exerting an immunomodulatory effect.

### 2.3. Effects of HCMP on the Growth of RAW 264.7 Macrophages

**Figure 2:**
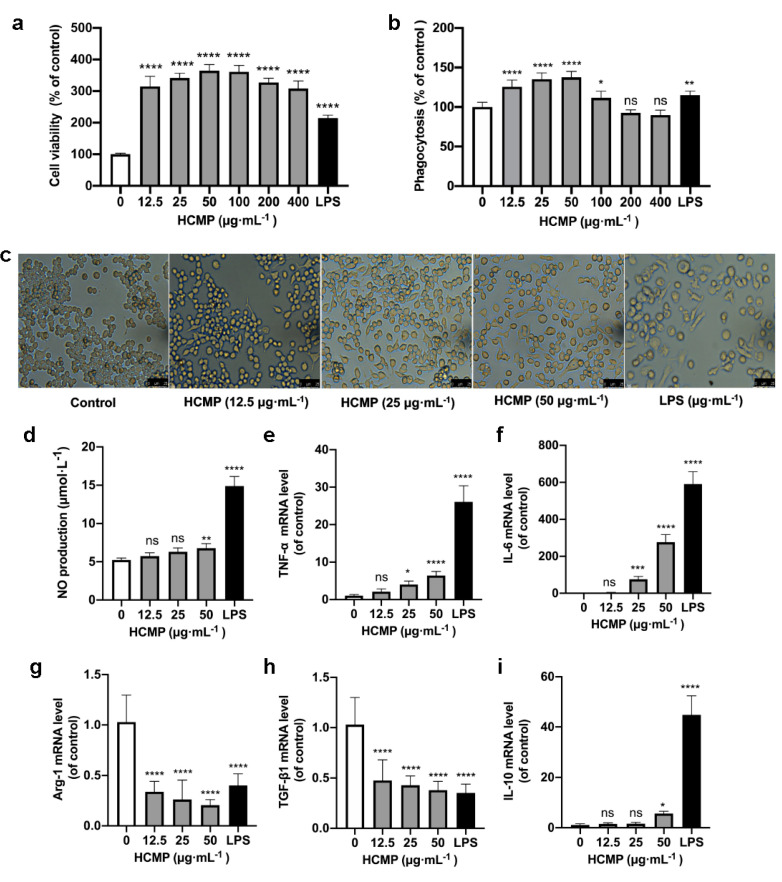
HCMP inducing RAW 264.7 macrophage activation. (**a**) The effects of HCMP on RAW 264.7 cell viability after cells were treated with various concentrations of HCMP for 24 h at 37 °C and 5% CO_2_. (**b**) The effects of HCMP on phagocytosis of RAW 264.7 macrophages after cells were treated with various concentrations of HCMP for 24 h at 37 °C and 5% CO_2_. (**c**) Microscopic images of morphological changes at 200× magnification after cells were treated with HCMP (50 μg·mL^−1^) or LPS (1 μg·mL^−1^) for 24 h at 37 °C and 5% CO_2_. The effects of HCMP on inflammation-related molecules in RAW 264.7 macrophages after cells were treated with various concentrations of HCMP for 6 or 24 h at 37 °C and 5% CO_2_: (**d**) NO production (μmol·L^−1^) for 24 h; the mRNA expression of (**e**) *TNF-α*, (**f**) *IL-6*, (**g**) *Arg-1*, (**h**) *TGF-β1* and (**i**) *IL-10* normalized to *β-actin*, for 6 h. ns, not significant; * *p* ≤ 0.05; ** *p* ≤ 0.01; *** *p* ≤ 0.001; and **** *p* ≤ 0.0001 versus control.

Cell viability assessment by the CCK-8 demonstrated that, compared to the control group, HCMP ranging from 12.5 to 400 μg·mL^−1^ notably enhanced the cell survival rate of RAW 264.7 macrophages (Figure 2a; *p* ≤ 0.0001). Within 12.5 to 400 μg·mL^−1^, HCMP exhibited no toxicity on RAW 264.7 macrophages and promoted the proliferation of RAW 264.7 macrophages. The highest cell activity reached 364.5% at 50 μg·mL^−1^ of HCMP. As the positive control, LPS (1 μg·mL^−1^) significantly stimulated cell proliferation (Figure 2a; *p* ≤ 0.0001).

### 2.4. HCMP Inducing the Activation of RAW 264.7 Macrophages

Phagocytosis is an initial and pivotal step for effectively eliminating pathogenic agents by macrophages. To investigate whether HCMP could activate RAW 264.7 macrophages, the phagocytosis of RAW 264.7 macrophages was detected by the neutral uptake assay. The results showed that HCMP at concentrations ranging from 12.5 to 100 μg·mL^−1^ markedly facilitated the phagocytosis of RAW 264.7 macrophages (Figure 2b; at 12.5–50 μg·mL^−1^ *p* ≤ 0.0001; at 100 μg·mL^−1^ *p* ≤ 0.05). HCMP exhibited no significant effect on the phagocytosis at 200 and 400 μg·mL^−1^ compared with the control group. These data indicate an enhanced immune activation effect of HCMP on RAW 264.7 macrophages. Cell viability and phagocytosis of RAW 264.7 cells reached the maximum level at 50 μg·mL^−1^ HCMP (Figure 2a,b).

Next, RAW 264.7 macrophage morphology was analyzed after cells were treated with HCMP (50 μg·mL^−1^) or LPS (1 μg·mL^−1^) under the microscope (200×) (Olympus, Tokyo, Japan). The control group cells maintained a small and round shape. In contrast, HCMP-treated cells exhibited an elongated spindle shape (Figure 2c). As the positive control, LPS induced cell enlargement, irregularity, and fragmentation (Figure 2c). A study by Zhu et al. [19] demonstrated that the immunomodulatory polysaccharide (AGPp) from *Arca granosa* Linnaeus triggered RAW 264.7 macrophage activation, leading to the formation of pseudopods. In our study, we noted similar changes in macrophage morphology in RAW 264.7 cells and HCMP-improved phagocytosis in RAW 264.7 cells. Thus, it could be speculated that HCMP activates RAW 264.7 cells.

### 2.5. HCMP Inducing Production and mRNA Expression of Inflammation-Related Molecules in RAW 264.7 Macrophages

Activated macrophages release inflammation-related molecules such as NO, TNF-α, and IL-6, which play crucial roles in immune responses and host defense. NO serves as the signaling molecule associated with macrophage cytotoxic function and contributes to eliminating microbes and tumor cells [20,21]. Our results showed that HCMP induced an increase in NO production in a dose-dependent manner in RAW 264.7 macrophages, following a 24 h treatment period (Figure 2d; at 50 μg·mL^−1^ *p* ≤ 0.01). The increased production of NO suggests that HCMP may activate the bactericidal and tumoricidal activity of macrophages. NO was one of the most studied inflammation-related molecules in the studies of immune responses induced by natural polysaccharides. Wang et al. [22] found that RAW 264.7 cells treated with *Lepidium meyenii* Walp. polysaccharide increased NO production at 1000 μg·mL^−1^ (*p* ≤ 0.01). Liu et al. [23] found that RAW 264.7 cells treated with acetylated *Cyclocarya paliurus* polysaccharide at 25–100 μg·mL^−1^ increased the NO amount (*p* ≤ 0.01). The stimulation of macrophages to secrete NO could reflect the effects of HCMP on immune function.

TNF-α and IL-6 represent pro-inflammatory molecules for classically activated M1 macrophages [24,25], while Arg-1 and TGF-β1 represent anti-inflammatory molecules, characteristics of alternatively activated M2 macrophages [26]. Following HCMP treatment for 24 h, the transcriptional expression of inflammation-related molecules was determined. Data were indicative of a significant increase in the mRNA expression of pro-inflammatory cytokines *TNF-α* (Figure 2e; at 25 μg·mL^−1^ *p* ≤ 0.05; 50 μg·mL^−1^ *p* ≤ 0.0001) and *IL-6* (Figure 2f; at 25 μg·mL^−1^ *p* ≤ 0.001; 50 μg·mL^−1^ *p* ≤ 0.0001). TNF-α is known to be among the initial cytokines produced, subsequently stimulating the release of other cytokines and immune cells, thereby facilitating immune responses [27,28]. IL-6 is an important cytokine produced by activated macrophages, contributing to the pathogenesis of various inflammatory diseases and immunological disorders. The production and transcriptional expression of *IL-6* were increased in hydroxyethylated *Hericium erinaceus* polysaccharide [8] and sulfated polysaccharide from *Porphyra haitanensis* [29]. Additionally, HCMP exhibited an inhibitory effect on the mRNA expression of *Arg-1* and *TGF-β1* at 12.5–50 μg·mL^−1^ (Figure 2g,h; *p* ≤ 0.0001) in a dose-dependent manner. Bai et al. [30] found that *Polygonatum* polysaccharide induced macrophage M1 polarization by significantly upregulating the mRNA levels of *TNF-α*, while downregulating the mRNA levels of Arg-1 and *TGF-β1* (*p* ≤ 0.05). Moreover, it revealed that HCMP at 50 μg·mL^−1^ notably boosted the mRNA level of anti-inflammatory cytokine *IL-10* (Figure 2i; *p* ≤ 0.0001), indicating a potential regulatory effect. IL-10 is a pleiotropic cytokine, which plays significant roles in inflammation and immune responses and functions to adjust the intensity of the immune and inflammatory responses to the severity of destruction produced by a pathological condition or a pathogen [31,32]. Liu et al. [29] found that sulfated polysaccharide from *Porphyra haitanensis* (PHPS) promoted the released levels of NO, TNF-α, and IL-6, and mRNA levels of *iNOS*, *IL-6*, and *TNF-α*. At the same time, PHPS induced IL-10 production, playing a critical role in immunoregulation. It could be deduced that while inducing the expression of *IL-6* and *TNF-α*, HCMP also promoted the transcriptional expression of *IL-10* to adjust the intensity of the immune and inflammatory responses. 

In response to an immune challenge, macrophages become activated and produce cytotoxic and inflammatory mediators such as NO, TNF-α, and IL-6, contributing to nonspecific immunity [33]. The effects of HCMP on NO production and the mRNA expression of inflammation-related molecules further indicated that HCMP effectively activated RAW 264.7 macrophages. As the positive control, LPS significantly promoted NO production and the transcriptional expression of *TNF-α*, *IL-6*, and *IL-10*, and decreased the transcriptional expression of *TGF-β1* and *Arg-1* (Figure 2d–i; *p* ≤ 0.0001). As a result, HCMP exhibited its potential pro-inflammatory effects.

### 2.6. The Effects of HCMP on Inflammation-Related Molecules in an Inflammatory Environment

**Figure 3:**
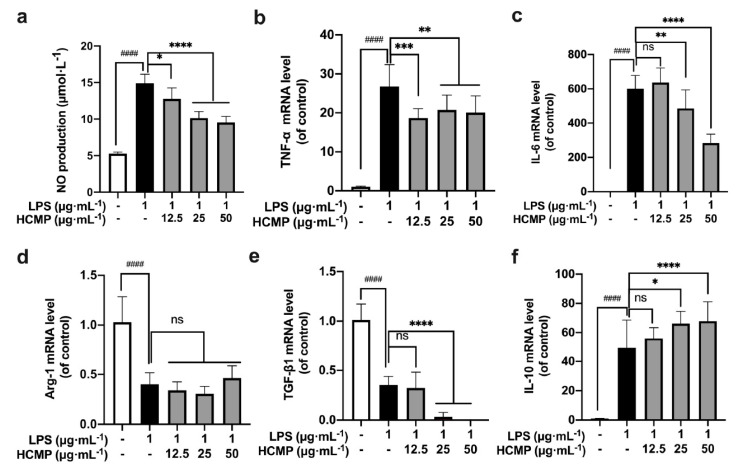
The effects of HCMP on inflammation-related molecules in LPS-induced RAW 264.7 macrophages. RAW 264.7 macrophages were treated with LPS (1 μg·mL^−1^) for 2 h, and then were subjected to various concentrations of HCMP (12.5, 25, 50 μg·mL^−1^) for 6 h or 24 h, at 37 °C and 5% CO_2_: (**a**) NO production (μmol·L^−1^) for 24 h; the mRNA expression of (**b**) *TNF-α*, (**c**) *IL-6*, (**d**) *Arg-1*, (**e**) *TGF-β1* and (**f**) *IL-10* normalized to *β-actin*, for 6 h. ns, not significant; * *p* ≤ 0.05; ** *p* ≤ 0.01; *** *p* ≤ 0.001; and **** *p* ≤ 0.0001 versus LPS group. #### *p* ≤ 0.0001 versus blank control.

To assess HCMP’s impact on the inflammatory environment, RAW 264.7 macrophages were treated with LPS (1 μg·mL^−1^) for 2 h, and then were subjected to various concentrations of HCMP (12.5, 25, 50 μg·mL^−1^) for 6 h. We found a notable increase in NO production (Figure 3a; at 1 μg·mL^−1^ of LPS, *p* ≤ 0.0001) and the transcriptional expression of *TNF-α* (Figure 3b; at 1 μg·mL^−1^ of LPS, *p* ≤ 0.0001) and *IL-6* (Figure 3c; at 1 μg·mL^−1^ of LPS, *p* ≤ 0.0001) in the LPS-induced RAW 264.7 cells compared to the control group, indicating the successful establishment of an inflammatory model by LPS. Next, HCMP treatment decreased NO production in a dose-dependent manner (Figure 3a; at 25 and 50 μg·mL^−1^ *p* ≤ 0.0001), and the mRNA expression of *TNF-α* (Figure 3b; at 25 and 50 μg·mL^−1^ *p* ≤ 0.01) and *IL-6* (Figure 3a; at 25 μg·mL^−1^ *p* ≤ 0.01; 50 μg·mL^−1^ *p* ≤ 0.0001) in LPS-induced cells, indicating that HCMP alleviated the inflammation induced by LPS. Immune factors are predominant in the pathogenesis of inflammatory bowel disease [34]. Anti-inflammatory and immunosuppressive treatments are known for reducing and limiting the damage caused by inflammatory bowel disease. Chen et al. [34] demonstrated that *Hericium erinaceus* extracts decreased the expression levels of pro-inflammatory cytokines such as *IL-1α*, *IL-2*, *IL-8*, and *TNF-α*, and inhibited the nuclear factor-kappa B (NF-κB) signaling pathway to suppress inflammation directly. In addition, a further increase in the mRNA expression of *IL-10* upon HCMP treatment was observed (Figure 3f; at 25 μg·mL^−1^ *p* ≤ 0.05; at 50 μg·mL^−1^ *p* ≤ 0.0001), which might function to adjust the intensity of the immune and inflammatory responses. Compared with the LPS group, HCMP did not cause further changes in the mRNA expression of *Arg-1* (Figure 3d; at 12.5–50 μg·mL^−1^ not significant) and further reduced the mRNA expression of *TGF-β1* (Figure 3e; at 25 μg·mL^−1^ and 50 μg·mL^−1^ *p* ≤ 0.0001). These findings highlight the anti-inflammatory properties of HCMP.

When stimulated by a foreign substance, macrophages promptly respond by differentiating into M1 and M2 macrophages. This polarization phenomenon allows M1 macrophages to upregulate immune responses while M2 macrophages downregulate excessive immune reactions, thereby maintaining immune system balance [35]. The dynamic polarization between M1 and M2 endows macrophages to swiftly adapt to various stimuli and environmental cues [36,37]. For example, necrotizing enterocolitis could potentially be improved by diminishing M1 macrophage activity while augmenting M2 polarization [38]. Conversely, the transition of M2 macrophages to M1 is considered a favorable therapeutic intervention in various tumor immune microenvironments [39,40]. Based on these results, HCMP attenuated the expression of inflammation-related molecules in LPS-induced inflammatory models, diminishing M1 macrophage activity while augmenting M2 macrophage activity. HCMP evidenced the capability to balance M1/M2 macrophages by regulating pro-inflammatory and anti-inflammatory-related molecules in the immune responses.

### 2.7. HCMP Activating MAPK Signaling Pathway

**Figure 4:**
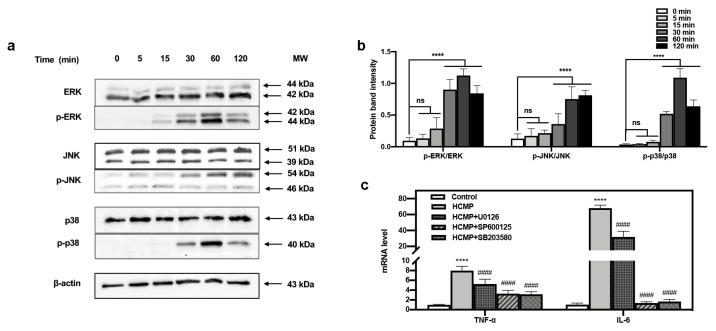
Effects of HCMP on the MAPKs activation in RAW 264.7 macrophages. (**a**) HCMP increased phosphorylation of ERK, JNK, and p38 evaluated by Western blotting analysis. β-actin protein served as the loading control. (**b**) The phosphorylation intensity analysis of ERK, JNK, and p38. (**c**) The mRNA expression of *TNF-α* and *IL-6* in RAW 264.7 macrophages was determined after treatment with HCMP (50 μg·mL^−1^) combined with MAPK signaling pathways inhibitors (U0126 for ERK, SP600125 for JNK, SB203580 for p38) for 6 h. ns, not significant; **** *p* ≤ 0.0001 versus control; #### *p* ≤ 0.0001 versus HCMP.

The defense mechanism of macrophages against external stimuli can be traced back to the PRRs on the surface of cells [9,41], which recognize and bind their respective ligands and trigger downstream signaling pathways, leading to the transcriptional activation and expression of inflammation-related molecules [42,43,44]. The MAPK pathway functions in many physiological processes—for instance, cell growth, apoptotic cell death, immune defense, etc. [9]. The functions mediated by MAPKs often involve phosphorylation events [19,20] where phosphorylated MAPKs (p-MAPKs) are translocated to the nucleus, facilitating the transcription of MAPK-regulated genes [43]. Our investigation delved into the immune regulatory mechanism of HCMP. The impact of HCMP on the phosphorylation of MAPK proteins was assessed via Western blotting. We have tracked the dynamic activation of MAPKs induced by HCMP (50 μg·mL^−1^). ERK, JNK, and p38 phosphorylation gradually increased in 30 min, and reached the maximal level at 60 min (JNK at 120 min); after 120 min, they returned to the basal level (Figure 4b). Hsu et al. [45] found that extract of Reishi polysaccharide (EORP) induced the immunomodulating activities of mouse spleen cells through the MAPK signaling pathway, and Western blotting showed that EORP improved JNK and p38 phosphorylation at 10 min and reached the maximal level at 20 min, while after 120 min, JNK and p38 phosphorylation returned to the basal level. At 30, 60, and 120 min, the band imprints of p-ERK, p-JNK, and p-p38 MAPKs groups were deepened in the protein band diagram at the specified time points (Figure 4a,b; at 30, 60, 120 min, *p* ≤ 0.0001), which indicated that HCMP raised the phosphorylation degree of ERK, JNK, and p38 MAPKs.

**Figure 5:**
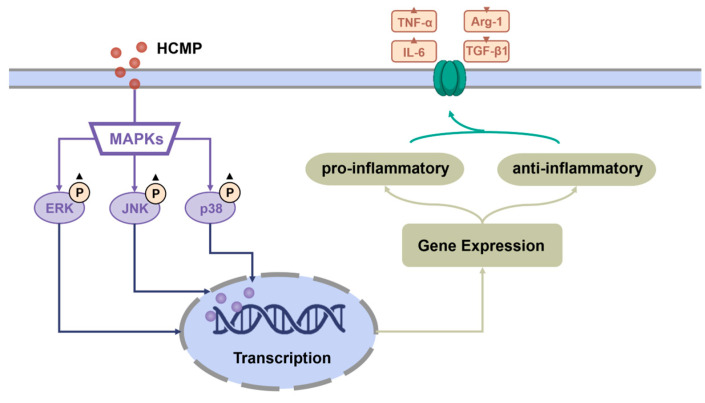
Potential immunomodulatory mechanism of HCMP activating RAW 264.7 macrophages.

Moreover, to ascertain the involvement of the MAPK signaling pathway in the activation of RAW 264.7 cells by HCMP, the effects of inhibitors of U0126 (ERK1/2 inhibitor), SP600125 (SAPK/JNK inhibitor), and SB203580 (p38 inhibitor) on the mRNA expression of *TNF-α* and *IL-6* induced by HCMP were checked. Data depicted that the inhibitors targeting MAPKs significantly attenuated the mRNA levels of *TNF-α* and *IL-6* compared with the HCMP-treated groups (Figure 4c; *p* ≤ 0.0001). These findings demonstrate that the inhibition of the MAPK pathway suppressed the mRNA expression of pro-inflammatory cytokines induced by HCMP. Therefore, these findings indicate that HCMP regulated the mRNA expression of inflammation-related molecules through activating the MAPK pathway in RAW 264.7 macrophages in response to external stimuli (Figure 5).

Due to the lack of specificity on the biological activity, and the predicted negative impact of LPS on the growth and performance of poultry, LPS should probably be reserved for experimental use and not for commercial use [46]. Researchers have focused more attention on developing novel immunopotentiator drugs with demonstrated biological specificity and little or no adverse effect on the growth and performance of the host. HCMP might be a candidate to replace LPS as a novel immunopotentiator drug.

## 3. Materials and Methods

### 3.1. Materials

The fruiting bodies of *C. militaris* were purchased locally. DEAE-Sephadex A-25 and Sephadex G-100 were obtained from GE Healthcare (Fairview, CT, USA). Dulbecco’s modified Eagle’s medium (DMEM), fetal bovine serum (FBS), and antibiotics were sourced from GIBCO (Grand Island, NE, USA). Lipopolysaccharide (LPS), cell counting kit-8 (CCK-8) assay kit, and neutral red solution were acquired from Sangon Biotech (Shanghai, China). The horseradish peroxidase (HRP)-conjugated goat, anti-mouse IgG, and anti-rabbit IgG were acquired from Yeasen (Shanghai, China). The SYBR qPCR Master Mix for RT-qPCR was purchased from Vazyme (Nanjing, China). The BCA protein extraction kit was purchased from Yeasen (Shanghai, China). The phospho-ERK1/2, ERK1/2, phospho-JNK1/2/3, JNK1/2/3, phospho-p38, p38, and β-actin antibodies were obtained from Abcam (Shanghai, China). The RNeasy Mini Kit and PrimeScript™ RT Master Mix were sourced from Tiangen (Beijing, China).

### 3.2. Isolation and Purification of C. militaris Polysaccharides

The crude polysaccharide was extracted from *C. militaris* using hot water extraction and the ethanol precipitation method. DEAE-Sephadex A-25 and Sephadex G-100 columns were employed for the purification of crude polysaccharides [12]. Firstly, the fruiting bodies of *C. militaris* were ground into powders; the powders were dissolved in distilled water (1:20, *w*/*w*) and extracted at 90–95 °C for 3 h, and the supernatant was concentrated and subjected to precipitation using 80% ethanol (*v*/*v*) to obtain crude polysaccharide. Following this, the crude polysaccharide was deproteinized and decolorized after treatment with Sevage reagent (chloroform:n-butanol = 4:1, *v*/*v*), H_2_O_2_ (50 °C) and fluid dialysis, respectively. Next, the solution was loaded onto a DEAE-Sephadex A-25 cellulose column (2.6 × 60 cm), eluted by NaCl (0, 0.1, 0.2, 0.3, 0.4, 0.5 mol·L^−1^) to collect polysaccharide fractions at the peak of the elution sequentially. Subsequently, Fraction I was further eluted by Sephadex G-100 dextran gel column (1.6 × 40 cm) by distilled water. Two elution components were collected, freeze-dried, and stored at − 20 °C, respectively. Since the Mw of the first elution component was relatively higher than the latter one, the first component eluted from Fraction I was named HCMP. The polysaccharide content of HCMP was determined using the phenol-sulfuric acid method [47].

### 3.3. Mw Determination of HCMP

The Mw distribution analysis was conducted using gel permeation chromatography (GPC) (Agilent 1200, Agilent, Santa Clara, CA, USA), which was equipped with an Agilent refractive index detector and an Agilent PL aquagel-OH MIXED-H column (300 × 7.5 mm, 8 µm) [13]. The mobile phase NaNO_3_ (0.1 M) went at a constant flow rate of 0.6 mL/min and 35 °C. A total of 100 μL of the sample (20 mg·mL^−1^) was loaded to detect the results. The Mw of HCMP was determined based on this standard curve, which was set up by plotting the logarithm of different Mw of dextran standards against their respective retention times.

### 3.4. Monosaccharide Composition Determination of HCMP

Monosaccharide composition was detected using a gas chromatograph spectrometer (GC, Trace1300-ISQ7000, Thermo Fisher Scientific, Waltham, MA, USA) equipped with an HP-5 capillary chromatographic column (30 mm × 0.32 mm, 0.25 µm) and FID detector [48]. Initially, 10 mg of HCMP was accurately weighed into an ampoule bottle and dissolved by 2 mL of trifluoroacetic acid (2 mol·L^−1^). The mixture was subsequently sealed and held at 105 °C for 8 h. The resulting hydrolysate was evaporated to dryness using a nitrogen-blowing instrument, and methanol was added in small increments multiple times and dried via blowing. Then, 10 mg of hydroxylamine hydrochloride and 0.5 mL of pyridine were introduced for a reaction at 90 °C for 30 min, followed by further drying via blowing. The resulting product was dissolved in trichloromethane for GC analysis. The sample inlet temperature was set at 250 °C and the temperature gradient procedure was conducted as follows: an initial temperature of 80 °C, gradually increased to 170 °C at a rate of 20 °C·min^−1^, held for 2 min, further increased to 240 °C at a rate of 2.5 °C·min^−1^, and finally increased to 280 °C at a rate of 10 °C min^−1^, maintaining for 6.5 min. Helium served as the carrier gas at a flow rate of 1.0 mL·min^−1^, and 0.5 μL of the sample was loaded.

### 3.5. Culture of RAW 264.7 Macrophages

The mice mononuclear RAW 264.7 macrophages were sourced by the Cell Bank of Typical Culture Preservation Committee of Chinese Academy of Sciences (Shanghai, China). RAW 264.7 macrophages were cultured and maintained in DMEM, supplemented with 10% FBS and 1% antibiotics in an environment of 5% CO_2_ at 37 °C.

### 3.6. Cell Viability Assay

HCMP’s impact on the cell viability of RAW 264.7 macrophages was assessed using CCK-8 assay. RAW 264.7 macrophages (4 × 10^5^ cells/mL) were plated in 96-well plates and allowed to incubate for 24 h at 5% CO_2_ and 37 °C, and subjected to various concentrations of HCMP (12.5–400 μg·mL^−1^) for an additional 24 h. Subsequently, the cells were incubated with 5 μL of CCK-8 for 2 h. The absorbance at 450 nm was recorded using a microplate reader (BioTek, Winooski, VT, USA). LPS (1 μg·mL^−1^) was employed as a positive control. According to the manufacturer’s recommendation, the commonly used concentration range for positive control LPS in treating cells was 0.01 to 1 μg·mL^−1^, with the most common concentration 0.1 μg·mL^−1^, while in most studies of LPS-induced macrophage inflammation models, the concentration of LPS was usually 1 μg·mL^−1^ [49,50]. In this study, we investigated both the pro-inflammatory and anti-inflammatory activity of HCMP, and the concentration of LPS used as a positive control was selected as 1 μg·mL^−1^ for simultaneous control.

### 3.7. Cell Phagocytosis Assay

To measure the phagocytosis capability of RAW 264.7 macrophages induced by HCMP, the neutral red uptake assay was employed [41]. RAW 264.7 macrophages (4 × 10^5^ cells/mL) were plated in 96-well plates and incubated for 24 h at 5% CO_2_ and 37 °C, and then subjected to various concentrations of HCMP (12.5–400 μg·mL^−1^) for 24 h, followed by treatment with neutral red solution (2 g·L^−1^) for 30 min. After this incubation, cells were washed with PBS twice and cracked with lysis buffer (ethanol: acetic acid = 1:1, *v*/*v*), then shaken at room temperature for 2 h. The absorbance at 540 nm was recorded using a microplate reader (BioTek, Winooski, VT, USA).

### 3.8. Cell Morphology Assessment

RAW 264.7 macrophages (4 × 10^5^ cells/mL) were plated in 12-well plates and incubated for 24 h at 5% CO_2_ and 37 °C, and then subjected to HCMP (50 μg·mL^−1^) or LPS (1 μg·mL^−1^) for 24 h. The morphology of RAW 264.7 cells was assessed using a light microscope (Olympus, Tokyo, Japan).

### 3.9. NO Assay

The quantification of NO production was conducted following the manufacturer’s protocol of the NO assay kit. RAW 264.7 macrophages (4 × 10^5^ cells/mL) were plated in 96-well plates and incubated for 24 h at 5% CO_2_ and 37 °C, and then subjected to various concentrations of HCMP (12.5–50 μg·mL^−1^) for 24 h. After the incubation, cell culture supernatant was collected for NO production analysis. Briefly, 50 μL of the supernatant was mixed with equal volumes of Griess reagent I and Griess reagent II for the reaction, according to the manufacturer’s protocol [51]. The absorbance at 540 nm was recorded using a microplate reader (BioTek, Winooski, VT, USA). LPS (1 μg·mL^−1^), which served as a reference standard.

### 3.10. RT-qPCR Assay

RAW 264.7 macrophages (2 × 10^5^ cells/mL) were plated in 12-well plates and incubated for 24 h at 5% CO_2_ and 37 °C, and then subjected to various concentrations of HCMP (ranging from 12.5 to 50 μg·mL^−1^) for 6 h. After the treatment, the cells were collected, and the total RNA was extracted using an RNeasy Mini Kit. Following RNA extraction, cDNA was synthesized out of 1 μg of RNA using the PrimeScript^TM^ RT-PCR kit. The reaction conditions involved incubation at 37 °C for 5 min, followed by a subsequent step at 85 °C for 5 s. To evaluate the effects of HCMP on the mRNA expression of inflammatory genes, an RT-qPCR assay was conducted based on the cDNA product (diluted 50 times) to determine the mRNA expression levels. For qRT-PCR assay, the reaction mixture included 5 μL 2× AceQ Universal SYBR qPCR Master Mix, 0.2 μL forward primer and 0.2 μL reverse primer, 0.2 μL, 2 μL cDNA, and 2.6 μL dH_2_O [52]. The program and conditions of PCR were set as follows: starting denaturation at 95 °C for 5 min, annealing and extension at 60 °C for 30 s for 35 cycles, and a final extension step at 72 °C for 5 min. β-actin was employed for an internal control. The expression amount of each target cytokine was calculated by the 2^−ΔΔ*C*t^ method. The primer sequences in this study are listed in Table 1. 

### 3.11. Western Blotting

RAW 264.7 cells (1 × 10^6^ cells /mL) were incubated for 24 h and subjected to HCMP (50 μg·mL^−1^) at 5, 15, 30, 60 and 120 min. Total cell lysates were obtained using a BCA assay kit. Briefly, cells were lysed using cell lysis buffer, and the lysates were concentrated at 14,000× *g* for 20 min at 4 °C to obtain the total protein supernatant. Denaturation of all protein samples was denatured using 5 × protein loading buffer at 100 °C for 5 min. In total, 20 μg of samples was loaded onto sodium dodecyl sulfate polyacrylamide gel electrophoresis (SDS-PAGE) for 90 min to separate the protein bands, and was immediately transferred to the polyvinylidene difluoride fluoride (PVDF) membrane (Millipore Sigma, Burlington, MA, USA) via wet transfer (100 V for 70 min). Then, the PVDF membrane was washed 3 times with TBST (Tris 10 mmol·L^−1^, NaCl 150 mmol·L^−1^, Tween-20 (0.1%, *v*/*v*) and blocked with TBST containing 5% non-fat milk for 1 h at room temperature. Then, PVDF membranes were gently shaken with the corresponding primary antibody dilution overnight at 4 °C. After that, membranes were further reacted with the secondary antibody (goat anti-mouse or anti-rabbit IgG) for 2 h. The ChemiDoc XRS imaging system (Bio-Rad, Richmond, CA, USA) was used for Western blotting detection with ECL [53]. Band intensities were quantified using Image Lab Software (6.0.1).

### 3.12. Validation of HCMP Inducing MAPK Signaling Pathways

In order to further confirm the mechanism involved in HCMP-induced macrophage activation, ERK1/2 inhibitor U0126 (50 μmol·L^−1^), SAPK/JNK inhibitor SP600125 (30 μmol·L^−1^), and p38 MAPK inhibitor SB203580 (25 μmol·L^−1^) were used in RAW 264.7 cells, respectively. After that, cells were exposed to HCMP (50 μg·mL^−1^) for 6 h, and the mRNA level of *TNF-α* and *IL-6* was measured by RT-qPCR.

### 3.13. Statistical Analysis

The data obtained were analyzed using Graph Pad Prism 8 and expressed as mean ± standard deviation (SD). All experiments were independently repeated triplicate to ensure reliability and reproducibility. Statistical analysis was performed using a one-way analysis of variance (ANOVA). *p* ≤ 0.05 was considered to be statistically significant (ns, not significant; * *p* ≤ 0.05; ** *p* ≤ 0.01; *** *p* ≤ 0.001; and **** *p* ≤ 0.0001).

## 4. Conclusions

This study analyzed the structural feature and immunomodulatory activity of the bioactive polysaccharide HCMP isolated from the fruiting bodies of *C. militaris*. HCMP is a novel high-Mw polysaccharide (6.18 × 10^5^ Da), beyond the range of Mw previously found in *C. militaris* polysaccharides, which might complement the existing research. In addition, HCMP comprised a higher percentage of galactose, glucose, and mannose, endowing HCMP likely to be recognized by macrophage surface receptors to trigger specific signaling pathways. HCMP effectively acted with ERK, JNK, and p38 MAPK and activated the MAPK signaling pathway, thereby regulating the transcriptional expression of inflammation-related molecules and triggering immune responses in RAW 264.7 macrophages. Moreover, HCMP exhibited the ability to avoid excessive inflammation to promote health. Overall, HCMP, a novel polysaccharide sourced from the edible and medicinal mushroom, may be a potential immunomodulatory agent to enhance human immunity. Still, the relevant endotoxin and animal experiments should be carried out before the clinical application.

## Figures and Tables

**Table 1:** The primer sequences of the investigated genes in an RT-qPCR analysis.

Gene	Direction	Primer Sequence (5′→3′)
β-actin	Forward	ATCGTGCGGGACATCAAGG
	Reverse	TCGTTGCCGATGGTGATGAC
TNF-α	Forward	GGGGATTATGGCTCAGGGTC
	Reverse	CGAGGCTCCAGTGAATTCGG
IL-6	Forward	CATGTTCTCTGGGAAATCGTGG
	Reverse	AACGCACTAGGTTTGCCGAGTA
Arg-1	Forward	CAGAAGAATGGAAGAGTCAG
	Reverse	CAGATATGCAGGGAGTCACC
TGF-β1	Forward	ACAGCACCAATTGTCCAAGTTTC
	Reverse	CGGTGCATGCATAGCCTTGT
IL-10	Forward	CCAAGCCTTATCGGAAATGA
	Reverse	TCCTGAGGGTCTTCAGCTTC

## Data Availability

Data are contained within the article.
